# Marital transition and cognitive function among older adults: the korean Longitudinal Study of Aging (2006–2020)

**DOI:** 10.1186/s12877-022-03697-x

**Published:** 2022-12-28

**Authors:** Nataliya Nerobkova, Yu Shin Park, Jaeyong Shin, Eun-Cheol Park

**Affiliations:** 1 Department of Public Health, Graduate School, Yonsei University, Seoul, Republic of Korea; 2 Institute of Health Services Research, Yonsei University, Seoul, Republic of Korea; 3 Department of Preventive Medicine, Yonsei University College of Medicine, 50 Yonsei-to, Seodaemun-gu, Seoul, 03722 Republic of Korea; 4 Department of Policy Analysis and Management, College of Human Ecology, Cornell University, Ithaca, NY USA

**Keywords:** Marital transition, Cognitive function, MMSE, Dementia, Longitudinal study

## Abstract

**Background:**

Marital status has been suggested as an associated factor for cognitive impairment. The consequences of marital transitions are still understudied. This study evaluated the influence of marital transitions on cognitive function using longitudinal, nationwide data of Korean older adults.

**Methods:**

This research comprised a longitudinal sample of older adults aged ≥ 45 years old, drawn from the Korean Longitudinal Study of Aging (2006–2020). Marital transition was determined through the biennial assessment of change in marital status; cognitive function was measured using the Korean version of the Mini-Mental State Examination. We employed general estimating equations to assess the temporal effect of marital transition on cognitive function.

**Results:**

Compared to married individuals, the odds ratios (ORs) of cognitive decline were higher in not married and transitioned out of marriage participants: men (OR 1.32, 95% confidence interval (CI) 0.96–1.82; OR 1.42, 95% CI 0.90–2.24), women (OR 1.21, 95% CI 1.03–1.42; OR 1.20, 95% CI 1.01–1.52), respectively, despite the findings being not statistically significant in men. The participants who transitioned out of marriage over time also showed greater ORs for mild cognitive impairment: men (OR 1.39, 95% CI 0.79–1.87), women (OR 1.33, 95% CI 1.05–1.80), and dementia: men (OR 1.60, 95% CI 0.85–1.99), women: (OR 1.49, 95% CI 1.20–2.19).

**Conclusion:**

Marital transition is found to be associated with cognitive function decline. Not-married individuals and those who became divorced or widowed were associated with the risk of cognitive function decline. Particular attention should be paid to these marital transitioned groups.

**Supplementary Information:**

The online version contains supplementary material available at 10.1186/s12877-022-03697-x.

## Background

With the increasing age of the population worldwide [1, 2], focus and efforts have been shifted to tackling the burden that comes along with an aging demographic [3]. One of the emerging co-occurring consequences is cognitive impairment, which has become a tremendous public health concern [4]. Cognitive impairment and its intensified form, dementia, induce socioeconomic burden by causing poor quality of life [5, 6], prolonged hospital stays [7], and increased mortality rates [8–10].

Recently, researchers have devoted strenuous efforts to identify the risk factors for mild cognitive impairment (MCI) and dementia and designing preventive strategies and policies [11]. However, since its onset or early phase detection is complicated, these efforts are focused predominantly on adjoining behavioral and biological factors, with many patients remaining undiagnosed until they display serious functional impairment [12]. The current study was designed to identify the influence of a more comprehensive conceptualization of marital status and its transitions as a potential social risk factor on MCI development and its progression to dementia among older adults in South Korea (hereafter, Korea).

Married individuals are, on average, healthier than their unmarried counterparts. Marriage, as well as cohabitation, ensure protective health benefits by increasing the social support and stability of life, and reducing loneliness, the factors that are associated with cognitive abilities in older age [13]. Previous findings identified that men appear to receive more health benefits from marriage than women [14, 15]. Women tend to provide greater social support that husbands may gain from their wives than vice versa [16]. Moreover, wives motivate husbands to health regulatory and prevention behaviors [15]. Given the results of prior studies regarding gender differences in the association between marital status and health [17] and quality of life [18], we also aimed to examine gender-specific differences in marital transitions and their associations with cognitive function.

Among studies that considered the various factors associated with cognitive impairment and possible methods to prevent cognitive decline based on those factors, the relationship between marital status and cognitive impairment revealed relatively controversial evidence. The findings from the National Health and Aging Trends Study of respondents over 65 documented that those who were divorced or widowed were at higher risk of cognitive impairment, with no sex differences revealed [11]. However, another study by Liu et al. emphasized that all unmarried groups had higher odds for dementia, with greater ORs in men [19]. The population-based cohort study from Finland on the association between mid-life marital status and cognitive function revealed a specifically increased risk for widowed and divorced people compared with their single counterparts, claiming that living in a relationship might lead to cognitive and social challenges that, in turn, have a protective impact against cognitive impairment later in life [20]. However, due to the small number of sub-analyses, authors do not draw safe conclusions about possible sex differences.

In Korea, the findings were not entirely consistent as well. For example, the research by Lyu et al. of older adults over 45 years found no significant difference in the association between marital status and cognitive function [21]. In contrast, in the study by Bae et al., single, widowed, or divorced older individuals were at a higher risk of cognitive impairment [22]. However, no conclusions were made in terms of sex differences. Another study emphasized that psychosocial adjustment after a spousal loss was highly gendered [23]. Although, the study lacked information about divorce and never-married cases.

In recent decades, Korea has been experiencing rapid changes in marriage trends [24, 25]. Following the patterns of other Western and Asian countries, Korea has experienced a prompt decline in fertility since the 1960s, stimulated by socioeconomic development [26]. In addition, the number of older adults who are unmarried due to divorce or refrain from getting married has increased along with the aging of the population, and it is expected to increase continuously [27]. Researchers usually relate these changes to the second fertility transition, fast-growing gender equality, economic developments, expansion of higher education [25], and ideational changes [24].

Few studies have investigated the association between marital transition and cognitive impairment in Korea. However, examining such an association in a large sample and through a longitudinal study design would be a sound basis for preventing cognitive decline by putting extra control over vulnerable groups. The objective of the study is to address two research hypotheses: first, if there is an association between the time-varying marital transition and cognitive function, and second, if there is the role of sex in this association. A clear understanding of these relationships will provide substantial implications to policymakers for considering how to best support groups at risk and prevent poor mental health in later life. To this end, we aimed to evaluate changes in marital status with respect to the risk of cognitive impairment among the Korean older adult population based on a longitudinal study after adjusting for confounders that were assumed to affect cognitive function.

## Methods

### Data source and sample

**Fig. 1:**
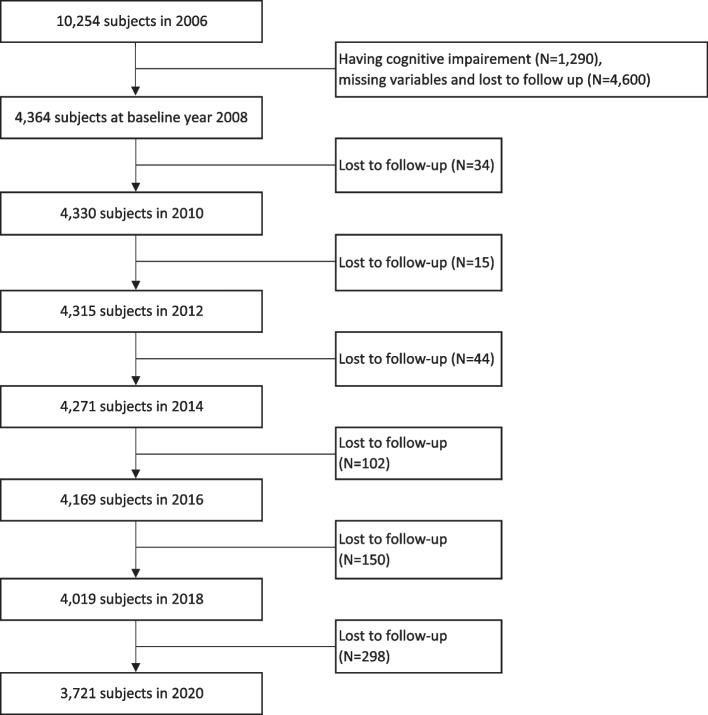
Flowchart of the study participants from 2006 to 2020

Our present study extracted data over 14 years from the 1st to 8th wave (2006 to 2020) of the Korean Longitudinal Study of Aging (KLoSA). Since its initiation in 2006, the Korea Labor Institute has been collecting regular panel survey data among the same sample of older adults aged more than 45 years from all regions around Korea. The total number of participants surveyed in 2008 was 8,688 (approximately 84.7% of the original 10,254 participants in 2006). The survey was conducted every even-numbered year starting from 2006 via computer-assisted interviews, mainly using the same survey categories with a sample retention rate of 63.3% in 2020. The survey gathered information on the respondents’ family background, demographics, family composition, health, employment, income, assets, subjective expectations, and subjective quality of life. Additional information about the survey can be obtained on the panel survey organization website (https://survey.keis.or.kr/klosa/klosa01.jsp). In the present study, we adopted survey data for a total of eight datasets. The exclusion criteria included cognitive impairment during the first wave (2006), missing information on employed variables, and individuals lost to follow-up, leading to the inclusion of 4,364 in 2008, 4,330 in 2010, 4,315 in 2012, 4,271 in 2014, 4,169 in 2016, 4,019 in 2018, and 3,721 participants in 2020. The selection process of participants is shown in detail in Fig. 1.

### Variables

The variable of interest, “marital transition,” was assessed as a time-varying covariate reflecting marital status at the time of the survey, with five categories: married or cohabiting, separated, divorced, widowed or missing (dispersed family), and never married. The lag function was employed to detect changes in marital status in the prior wave and the succeeding wave, following a 2-year gap. Thus, marital transitions were categorized into four groups: (1) married → married, (2) married → not married, (3) not married → married, and (4) not married → not married. Additionally, for the subgroup analysis, we considered the following categories: married → married, married→ divorced, married → widowed, not married → married, divorced → divorced, widowed → widowed, and not married → not married.

The outcome variable was identified by measuring cognitive function using the Korean version of the Mini-Mental State Examination (MMSE) score. The MMSE is a validated construct used to evaluate cognitive functioning in the Korean population [28, 29]. The construct consists of seven cognitive function categories, including time orientation, spatial orientation, registration, attention and calculation, recall, language, and visual construction domains [30]. These items comprise a compound score of 30 points, with higher scores indicating higher cognitive function. With an overall score of 30, the MMSE’s cut-off level for MCI is 23, and 19 is for the risk of dementia [29]. We used the total scores for analysis to reveal detailed results regarding the association.

Data on sociodemographic characteristics and health-related conditions were added as potential confounders in this study. Sociodemographic characteristics included sex, age (45–64, ≥ 65 years), educational level (middle school or below, high school or above), and income level per month (low, middle-low, middle-high, and high). Additionally, we considered the regions of residence (urban or rural areas), living arrangements (living alone or with another family member(s)), and a number of offspring (none, 1, and 2 or more). Limitations in activities of daily living were specified if the survey participants had difficulty performing any daily, necessary tasks, including getting dressed, face and hands washing, bathing, eating meals, leaving a room, and toilet usage. The chronic diseases considered in the current study included hypertension, diabetes mellitus, cancer, lung diseases, heart diseases, and cerebrovascular diseases. The number of comorbid diseases was arranged into three categories: none, 1, and ≥ 2 diseases.

In addition, we adjusted for handgrip strengths and subjective life satisfaction. Our data analysis excluded participants who did not manage to perform the test owing to physical conditions. Subjective life satisfaction was classified as bad, normal, and good.

### Statistical analysis

Lagged generalized estimating equation (GEE) analyses with an unstructured correlation structure were conducted and controlled for confounders to estimate the MMSE scores according to the 2-year transitions in marital status. All statistical analyses were conducted separately for men and women to examine gender-specific differences [31] regarding the diverse impact of marital transition on cognitive function. The GEE stands for repeated measure analysis of longitudinal panel and considers the relationship within the subject to generate odds ratios (ORs) and 95% confidence intervals (CIs), and the corresponding *p*-value. MMSE score distributions were additionally summarized as the mean and standard deviation (SD) for the baseline (2008) and last (2020) waves. A total of eight waves (2006–2020) were used for the analysis, and repeated measurements were carried out for each individual up to seven times. Two-year lagged changes in marital transition were calculated using marital status in the preceding and following waves (2006–2008, 2008–2010, 2010–2012, 2012–2014, 2014–2016, 2016–2018, and 2018–2020) with the lag function following a 2-year interval.

Furthermore, two subgroup analyses were performed to further reveal the relationship between marital transition and cognitive health; one looked at the specific features of marital status changes and other covariates on mental health. The other subgroup analysis was performed to explore the association between changes in marital status and MCI (MMSE score from 20 to 24) and dementia (MMSE score below 20). Findings were considered statistically significant with a *p*-value of < 0.05. All data analyses were performed using the SAS 9.4 software (version 9.4; SAS Institute Inc., Cary, NC, USA).

## Results

Table 1 summarizes the baseline characteristics of the study population stratified by sex. In total, 4,364 people were included in the baseline year (2,043 men and 2,321 women). The findings showed that the total percentage of women with cognitive impairment (MMSE scores below 24) was twice as high as that of men: 13.3% and 6.2%, respectively. Other covariates, such as age, region of residence, educational level, and household income, showed significant differences in the percentage of MMSE scores below and above 24 for both genders. The comparison of the mean MMSE scores in Wave 2 and Wave 8 is presented in Supplementary Table 1. In 2020 we noticed an overall decrease in the mean MMSE score in all groups. The most significant decrease was found in the Married → Not married group: men (mean 23.83, SD 5.22), women (mean 23.53, SD 5.33).

**Table 1:** General characteristics of the study population (baseline 2006→2008)

Variables	Cognitive function (MMSE score)													
	Male							Female						
	Total		< 24		≥ 24			Total		< 24		≥ 24		
	** N**	**%**	**N**	**%**	**N**	**%**	***P*** **-value**	**N**	**%**	**N**	**%**	**N**	**%**	***P*** **-value**
**Total *N* = 4 364**	**2043**	**100.0**	**127**	**6.2**	**1916**	**93.8**		**2321**	**100.0**	**309**	**13.3**	**2012**	**86.7**	
**Marital status**							0.1372							< 0.0001
Married → Married	1918	93.9	115	6.0	1803	94.0		1886	81.3	216	11.5	1670	88.5	
Married → Not married	13	0.6	0	0.0	13	100.0		25	1.1	3	12.0	22	88.0	
Not married → Married	2	0.1	0	0.0	2	100.0		1	0.0	0	0.0	1	100.0	
Not married → Not married	110	5.4	12	10.9	98	89.1		409	17.6	90	22.0	319	78.0	
**Age**							< 0.0001							< 0.0001
45–64	1377	67.4	47	3.4	1330	96.6		1701	73.3	130	7.6	1571	92.4	
≥65	666	32.6	80	12.0	586	88.0		620	26.7	179	28.9	441	71.1	
**Region**							0.0012							< 0.0001
Urban area	902	44.2	38	4.2	864	95.8		1096	47.2	99	9.0	997	91.0	
Rural area	1141	55.8	89	7.8	1052	92.2		1225	52.8	210	17.1	1015	82.9	
**Educational level**							< 0.0001							< 0.0001
Middle school or below	861	42.1	95	11.0	766	89.0		1489	64.2	285	19.1	1204	80.9	
High school or above	1182	57.9	32	2.7	1150	97.3		832	35.8	24	2.9	808	97.1	
**Household income**							< 0.0001							< 0.0001
Quartile 1 (low)	393	19.2	55	14.0	338	86.0		559	24.1	134	24.0	425	76.0	
Quartile 2	517	25.3	35	6.8	482	93.2		585	25.2	84	14.4	501	85.6	
Quartile 3	570	27.9	21	3.7	549	96.3		585	25.2	53	9.1	532	90.9	
Quartile 4 (high)	563	27.6	16	2.8	547	97.2		592	25.5	38	6.4	554	93.6	
**Living arrangement**							< 0.0001							< 0.0001
Living alone	54	2.6	8	14.8	46	85.2		209	9.0	44	21.1	165	78.9	
Living with a family member(s)	1989	97.4	119	6.0	1870	94.0		2112	91.0	265	12.5	1847	87.5	
**Number of offspring**							< 0.0001							< 0.0001
None	41	2.0	5	12.2	36	87.8		43	1.9	5	11.6	38	88.4	
1	146	7.1	5	3.4	141	96.6		161	6.9	12	7.5	149	92.5	
2	900	44.1	35	3.9	865	96.1		955	41.1	66	6.9	889	93.1	
≥3	956	46.8	82	8.6	874	91.4		1162	50.1	226	19.4	936	80.6	
**Chronic disease**							0.0056							< 0.0001
None	1269	62.1	63	5.0	1206	95.0		1476	63.6	151	10.2	1325	89.8	
1	562	27.5	42	7.5	520	92.5		635	27.4	112	17.6	523	82.4	
2 or more	212	10.4	22	10.4	190	89.6		210	9.0	46	21.9	164	78.1	
**ADL**							0.6462							0.7480
Normal	2033	99.5	126	6.2	1907	93.8		2311	99.6	308	13.3	2003	86.7	
Abnormal	10	0.5	1	10.0	9	90.0		10	0.4	1	10.0	9	90.0	
**Grip strength**							0.0056							< 0.0001
Normal	1901	93.0	98	5.2	1803	94.8		2124	91.5	254	12.0	1870	88.0	
Abnormal	142	7.0	29	20.4	113	79.6		197	8.5	55	27.9	142	72.1	
**Satisfaction of Life**							0.0002							< 0.0001
Bad	208	10.2	23	11.1	185	88.9		287	12.4	64	22.3	223	77.7	
Normal	1212	59.3	76	6.3	1136	93.7		1388	59.8	190	13.7	1198	86.3	
Good	623	30.5	28	4.5	595	95.5		646	27.8	55	8.5	591	91.5	

**Fig. 2:**
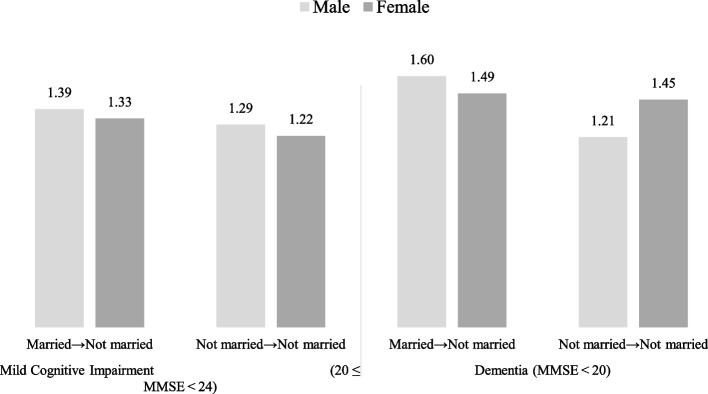
Results of the subgroup analysis of marital status and MMSE score

Table 2 depicts the findings of the GEE model results on the association between changes in marital status and the risk of cognitive impairment. Compared to those whose status remained Married → Married, we noted higher ORs for a Married → Not married transition (men: OR 1.42, 95% CI 0.90–2.24; women: OR 1.20, 95% CI 1.01–1.52) and the Not married → Not married group (men: OR 1.32, 95% CI 0.96–1.82; women: OR 1.21, 95% CI 1.03–1.42) in both genders, despite this association not being statistically significant in men. The Married → Not married and Not married → Not married groups also showed high ORs for MCI (score from 20 to 24): Married → Not married (men: OR 1.39, 95% CI 0.79–1.87; women OR 1.33, 95% CI 1.05–1.80), Not married → Not married (men: OR 1.29, 95% CI 0.78–1.75; women OR 1.22, 95% CI 1.10–1.47); and dementia (score below 20): Married → Not married (men: OR 1.49, 95% CI 1.20–2.19; women OR 1.49, 95% CI 1.20–2.19), Not married → Not married (men: OR 1.21, 95% CI 0.74–1.37; women OR 1.45, 95% CI 1.18–1.78), as illustrated in Fig. 2.

**Table 2:** Generalized linear model using the GEE with MMSE score in 2008–2020

Variables	MMSE score < 24			
	Male		Female	
	OR	95% CI	OR	95% CI
**Marital transition**				
Married → Married	1.00		1.00	
Married → Not married	1.42	(0.90 - 2.24)	1.20	(1.01- 1.52)
Not married → Married	0.77	(0.26 - 2.30)	0.47	(0.18 - 1.22)
Not married → Not married	1.32	(0.96 - 1.82)	1.21	(1.03 - 1.42)
**Age**				
45–64	1.00		1.00	
≥65	1.51	(1.27 - 1.80)	1.93	(1.69 - 2.19)
**Region**				
Urban area	1.00		1.00	
Rural area	1.24	(1.06 - 1.46)	1.42	(1.25-1.62)
**Educational level**				
Middle school or below	2.23	(1.88 - 2.65)	2.64	(2.20 - 3.17)
High school or above	1.00		1.00	
**Household income**				
Quartile 1 (low)	1.30	(1.05 - 1.60)	1.25	(1.05 - 1.48)
Quartile 2	0.89	(0.73 - 1.08)	0.98	(0.83 - 1.15)
Quartile 3	0.82	(0.68 - 0.98)	0.94	(0.81 - 1.10)
Quartile 4 (high)	1.00		1.00	
**Living arrangement**				
Living alone	0.85	(0.61 - 1.19)	1.12	(0.95 - 1.31)
Living with a family member(s)	1.00		1.00	
**Number of offspring**				
None	1.08	(0.89 - 1.32)	1.11	(0.91 - 1.34)
1	0.84	(0.60 - 1.18)	0.75	(0.55 - 1.03)
≥2	1.00		1.00	
**Chronic disease**				
None	1.00		1.00	
1	1.15	(0.98 - 1.36)	1.21	(1.07 - 1.38)
2 or more	1.45	(1.20 - 1.75)	1.38	(1.17 - 1.63)
**ADL**				
Normal	1.00		1.00	
Abnormal	2.84	(1.75 - 4.60)	1.99	(1.33 - 2.96)
**Grip strength**				
Normal	1.00		1.00	
Abnormal	1.90	(1.65 - 2.18)	1.78	(1.59 - 2.00)
**Satisfaction of Life**				
Bad	2.31	(1.88 - 2.83)	1.72	(1.47 - 2.02)
Normal	1.34	(1.16 - 1.54)	1.28	(1.14 - 1.43)
Good	1.00		1.00	
**Year**				
2008	1.00		1.00	
2010	1.27	(1.02 - 1.58)	1.04	(0.90 - 1.21)
2012	1.18	(0.95 - 1.48)	0.93	(0.81 - 1.08)
2014	1.71	(1.38 - 2.12)	1.18	(1.02 - 1.38)
2016	1.83	(1.48 - 2.27)	1.21	(1.04 - 1.41)
2018	2.58	(2.08 - 3.20)	1.30	(1.11 - 1.52)
2020	2.82	(2.23 - 3.56)	1.45	(1.23 - 1.69)

**Fig. 3:**
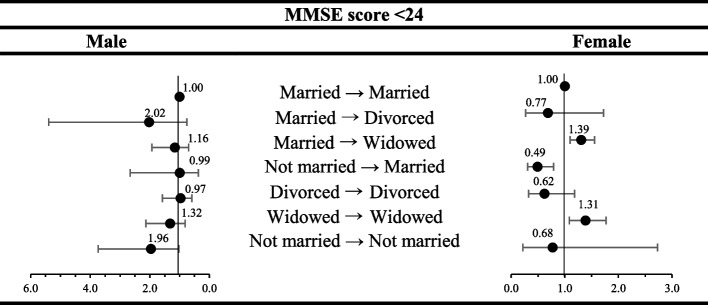
Results of the subgroup analysis of marital transition by sex

Figure 3 represents the longitudinal analysis results of the association between the specific features of the 2-year marital status changes and the risk of cognitive impairment. Given the results, we found higher odds in Married → Divorced (OR 2.02, 95% CI 0.76–5.39) and Not married → Not married (OR 1.96, 95% CI 1.03–3.73) groups in men, and in women Married → Widowed (OR 1.39, 95% CI 1.09–1.77) and Widowed → Widowed (OR 1.31, 95% CI 1.10–1.56) groups with the increased risk of MCI.

The independent subgroup analysis findings of the variables associated with the changes in marital status and cognitive impairment (MMSE scores below 24) are shown in Table 3. Though, subgroup analysis did not show statistical significance for men; for women, the Married → Not married and Not married → Not married groups, in particular, had the highest ORs estimate for respondents aged ≥ 65 years, compared to their younger counterpart: Married → Not married (OR 1.34, 95% CI 1.06–1.70) and Not married → Not married (OR 1.43, 95% CI 1.22–1.66). In addition, low educational level and rural region of residence were significantly associated with the increased risk of cognitive impairment in the same groups: Married → Not married (OR 1.32, 95% CI 1.04–1.66; OR 1.34, 95% CI 1.02–1.76) and Not married → Not married (OR 1.38, 95% CI 1.19–1.60; OR 1.52, 95% CI 1.27–1.82).

**Table 3:** Subgroup analysis using the GEE of MMSE score with marital transition in 2006–2020

Variables	MMSE score < 24													
	Male							Female						
	Married → Married	Married → Not married		Not married → Married		Not married → Not married		Married → Married	Married → Not married		Not married → Married		Not married → Not married	
	**OR**	OR*	**95% CI**	OR*	**95% CI**	OR*	**95% CI**	**OR**	OR*	**95% CI**	OR*	**95% CI**	OR*	**95% CI**
**Age**														
≥ 65	1.00	1.30	(0.77 -2.20)	1.28	(0.33 - 4.98)	1.30	(0.87 - 1.94)	1.00	1.34	(1.06 - 1.70)	0.49	(0.15 - 1.58)	1.43	(1.22 - 1.66)
**Region**														
Rural area	1.00	1.20	(0.70 - 2.05)	1.17	(0.46 - 2.98)	1.07	(0.73 - 1.58)	1.00	1.34	(1.02 - 1.76)	0.65	(0.26 - 1.63)	1.52	(1.27 - 1.82)
**Educational level**														
Middle school or below	1.00	1.82	(1.07 -3.09)	1.05	(0.30 - 3.76)	1.26	(0.86 - 1.83)	1.00	1.32	(1.04 - 1.66)	0.45	(0.16 - 1.26)	1.38	(1.19 - 1.60)

* Adjusted for other covariates

## Discussion

Cognitive function decline is a major geriatric health problem among older adults due to its aggravation with time [21]. The potential risk factors associated with cognitive function decline require careful and precise examination. Therefore, we investigated the association between marital transition and MCI development and progression to its intensified form, dementia, among Korean older adults over 45 years old.

The primary outcomes of our longitudinal study suggested that changes in marital status are associated with cognitive function decline; individuals who have never been married or who transitioned into divorce or widowhood showed the highest association with the risk of cognitive function decline compared to their married counterparts. Notably, the group of participants who transitioned out of marriage also showed a significant association with the intensified form of MCI, dementia, when compared with the “married” group. This finding supports the results of the previous studies where living alone as a non-married individual may be a risk for early-onset and late-onset dementia [32], and extends to transitioning out of marriage [19] as well.

Furthermore, we found that compared with other age groups, women over 65 years of age showed the strongest correlation between not being married and transitioning out of marriage and cognitive function decline. Therefore, our results suggest that this group was the most vulnerable. In addition, our findings showed that the association between marital status and cognitive health in women was more significant in those with low levels of education and those who resided in rural areas, suggesting that particular attention should be placed on these potentially vulnerable groups.

The etiology of the association between marital transition and cognitive function has not been fully established, although several possible explanatory mechanisms have been suggested. The first one lies in the stress process model [33] by understanding the pathways through which women appear to develop more mental health vulnerabilities than men as stressful life events and chronic stressors converge in the experience of marital status transitions [34, 35]. Supposing that mental health responses to marital change are the same for men and women in the short-term perspective and diverge at some point such that men recover at a more rapid rate than women. In that case, the issue of greater exposure of women to emerging secondary stressors such as economic hardship or single parenthood following an exit of marriage emerges as another explanation why women’s emotional reactivity to marital loss is more robust than that in men.

The concept of marriage could be another explanation of the relationship between marital transition and reduced cognitive function. Traditionally, married individuals are considered to be healthier both physically and mentally than their unmarried counterparts [14], and transition into marriage is also health enhancing [36, 37] through an increasing amount of socioeconomic resources, more frequent social interaction, a more extensive social network, and better social and emotional support [17, 38]. Notably, previous studies emphasized that men appeared to receive more health benefits from marriage than women [14, 34, 37]. On the contrary, women reported significantly larger increases in psychological distress than men when their marriages broke down (both divorced [35, 37] or widowed [39]).

Meanwhile, in the current study, statistical significance was primarily found in women; nonetheless, increased ORs were observed in men as well. Therefore, this is a noteworthy finding that requires attention. Remarkably, transitions out of marriage do not always undermine health and may, in some cases, enhance it [17]. Our observation that men’s transition to divorce or widowhood showed no statistically significant association with cognitive function decline could be supported by the earlier-mentioned model on the stress process [33]. Therefore, life transitions that are usually considered stressful events may, on the contrary, be stress-relieving [40], leading to the improvement of physical and mental health when they bring an end to another long-lasting disturbance. For older male adults, transitioning to being single may result in recovery from the negative health consequences of being in a strained and problematic marriage [17], which could lead to low statistical significance for them. Furthermore, in Korea, with a solid traditional gender role attitude in marriage [41] among older adults, men tend to experience less economic hardship [42] and have a higher chance of re-partnering [43] after transitioning out of marriage, lowering the chance of mental strain. Hence, in view of the inconsistency and given the observational nature of this study and the low rate of marital transition among men with the imprecise 95% CIs for some variables, the findings should be interpreted with caution.

The current study has several limitations. First, all the data was self-reported and collected via survey; thus, we cannot exclude the risk of biased results. Second, the data of those who did not answer the essential covariate questions were excluded, which may have caused the underestimation of cognitive function in the participants. Third, biological risk factors that might significantly affect adjustment variables were overlooked. Future data and analysis are required to fully discern the link between specific biological covariates and their impact on cognitive function with respect to marital transition. Finally, MMSE as a tool for screening cognitive function should be considered cautiously and supported with a neurological examination.

Nevertheless, the strengths of our study include the relatively large sample size and longitudinal design, with results being representative of the Korean older adult population over 45 years old. Our longitudinal analysis represents an improvement over cross-sectional models, which raise questions about selection and reverse causal order. The panel data we employ allow us to temporally order our analysis to reduce the probability that associations between marital status and cognitive function reflect its influence on the probability of becoming and remaining married. Another strength is that the study provides an in-depth view of marital transition and related to its risk of MCI and dementia progression. Hence, exploring the dynamics of change over time of marital status on cognitive function provides substantial implications for better control and supervision of vulnerable groups and emphasizes the need to develop integrated policies targeted toward the overall prevention measures for cognitive function decline. Additionally, since we used a standardized tool for measuring cognitive function, the findings provide a substantial basis for future studies. Finally, under the changing trends in marriage culture and the increasing age of the population, our study represents the most up-to-date data on the current topic.

## Conclusions and implications

In conclusion, the current study identified the relationship between marital transition and cognitive function among Korean older adults. Never-married individuals and those who transitioned into divorce or widowhood were associated with a higher risk of cognitive function decline and its progression to dementia compared to their married counterparts. The number of unmarried and divorced people in Korea is expected to increase continuously along with the aging of the population. Therefore, it is highly important to further explore the underlying mechanisms between the complex characteristics of marital relationships (marriage duration, quality) and their contribution to cognitive function. Our findings suggest that cognitive function assessment during routine medical check-ups and policies targeted at preventing cognitive impairment with particular attention paid to these potentially vulnerable groups are warranted.

## Supplementary Information

**Additional file 1.**

## Data Availability

The dataset supporting the conclusions of this article is available in the KLoSA repository, https://survey.keis.or.kr/klosa/klosa01.jsp.

## Abbreviations

OR: odds ratio
CI: confidence interval
GEE: generalized estimating equation
KLoSA: Korean Longitudinal Study of Aging
MMSE: Mini-Mental State Examination
MCI: mild cognitive impairment
SD: standard deviation.
