# Supplementary material for: Marital transition and cognitive function among older adults: the korean Longitudinal Study of Aging (2006–2020)
Source: BMC Geriatr. 2022 Dec 28;22:1003. doi: 10.1186/s12877-022-03697-x (PMC9798712; doi:10.1186/s12877-022-03697-x)
Supplement: Supplementary file 1 — Additional file 1. [file 12877_2022_3697_MOESM1_ESM.pdf]

Supplementary Table 1. Mean MMSE score in Baseline Wave 2 (2008) and Wave 8 (2020)

Variables	Cognitive function (MMSE score)																			
	Baseline Wave 2										Wave 8									
	Male										Female									
	N	%	Mean ± S.D.		P-value	N	%	Mean ± S.D.		P-value	N	%	Mean ± S.D.		P-value	N	%	Mean ± S.D.		P-value
**Total**	**2043**	**100.0**	**27.66**	**2.45**	**<.0001**	**2321**	**100.0**	**26.97**	**3.14**	**<.0001**	**1738**	**100.0**	**26.44**	**3.91**	**<.0001**	**1985**	**100.0**	**25.80**	**4.45**	**<.0001**
**Marital transition**					<.0001					<.0001					<.0001					<.0001
Married→ Married	1918	93.9	27.68	2.45		1886	81.3	27.16	2.96		1578	90.8	26.55	3.80		1428	71.9	26.34	4.00	
Married→ Not married	13	0.6	28.15	1.46		25	1.1	27.04	4.03		24	1.4	23.83	5.22		64	3.2	23.63	5.33	
Not married → Married	2	0.1	28.50	2.12		1	0.0	26.00	0.00		2	0.1	24.50	4.95		0	0.0	0.00	0.00	
Not married→ Not married	110	5.4	27.29	2.54		409	17.6	26.10	3.70		134	7.7	25.62	4.63		493	24.8	24.54	5.17	
